# Impact of Intraoperative Descemet Membrane Perforations on Deep Anterior Lamellar Keratoplasty Outcomes

**DOI:** 10.1155/joph/4101770

**Published:** 2025-07-17

**Authors:** Stephen Morgan, Ritika Mukhija, Mayank A. Nanavaty

**Affiliations:** ^1^Sussex Eye Hospital, University Hospitals Sussex NHS Foundation Trust, Brighton, UK; ^2^Brighton & Sussex Medical School, University of Sussex, Falmer, Brighton, UK

## Abstract

**Purpose:** To analyse the outcomes of deep anterior lamellar keratoplasty (DALK) in cases with intraoperative Descemet membrane (DM) perforation.

**Methods:** This is a literature review reporting outcomes of DALK with DM perforation. Studies where DALK was performed in the event of intraoperative DM perforation were included. Studies that did not separate analysis between those with and without DM perforation were excluded. The primary outcome was best-corrected distance visual acuity (BCVA). Secondary outcomes were endothelial cell density (ECD), graft survival, rejection rates, double anterior chamber and conversion to penetrating keratoplasty (PK). Data from the included studies were collated to compare the outcomes of DALK with intraoperative DM perforation vs. DALK without DM perforation.

**Results:** Eleven retrospective case series (357 eyes) were included. DM perforations were classified as micro- (*n* = 236) or macroperforations (*n* = 106). Mean weighted preoperative BCVA was 1.11 ± 0.36 logMAR and 1.13 ± 0.52 logMAR in perforation and nonperforation groups, respectively (*p*=0.53), improving to 0.35 ± 0.37 logMAR and 0.39 ± 0.07 logMAR at 12 months postoperatively (*p*=0.02). Graft rejection rates were 1.25% and 1.6% in the perforated and nonperforated groups, respectively, and primary graft failure rates were 4% and 3.74%, respectively. The mean postoperative ECD was 1662.41 ± 319.16 cells/mm^2^ in the perforation group. Amongst those cases with DM perforation, double anterior chamber requiring rebubbling occurred in 22.4% of cases, and conversion to PK was 4.23%.

**Conclusion:** DALK can achieve comparable long-term outcomes in the presence of DM perforation. Micro- and some macroperforations can often be managed without conversion to PK, with good long-term outcomes.

## 1. Introduction

Deep anterior lamellar keratoplasty (DALK) is a surgical procedure involving partial thickness corneal transplantation whilst sparing the host Descemet's membrane (DM) and endothelium, thereby having the primary advantages of being a ‘closed globe' surgery and a decreased risk of immune graft rejection [1]. However, the surgery is technically challenging, and DM perforation is the most feared intraoperative complication [1, 2].

Traditionally, the surgery was converted to a full-thickness penetrating keratoplasty (PK). However, there is now evidence that DALK can still be completed in the event of a DM perforation [2, 3]. This depends on the size and stage at which the perforation occurred, as well as the individual surgeon's experience. Huang et al. [4] and Seifelnasr et al. [5] defined microperforation as a small perforation that in general did not lead to consistent anterior chamber collapse (often a needle perforation), as compared to a macroperforation, in which a sizeable tear or gap in DM (usually 0.5 mm or more in length) resulted in persistent, complete collapse of the anterior chamber despite the use of air or balanced salt solution to reform the chamber [4, 5]. In addition to the surgeon's learning curve, irregular corneas, thin corneas and those with extensive stromal scarring have a higher risk of DM perforations [6]. Risk of perforation may also vary between techniques used (manual dissection vs. big bubble), with a potentially higher risk of perforation during type 2 bubble formation [7, 8]. While there are reports of successful management of various types of DM perforation, there is no consensus on their surgical management and approach may vary between surgeons and cases.

With this review, we aim to report the outcomes of DALK in cases of intraoperative Descemet's perforation.

## 2. Materials and Methods

### 2.1. Eligibility Criteria

Only peer-reviewed articles on human studies in the English language were included, and original articles, case reports and case series were included. We reviewed studies where DALK was performed in the event of intraoperative DM perforation. Studies that did not separate analysis between those with and without DM perforation were excluded. Studies that did not provide postoperative visual acuity data were also excluded. None of the studies were excluded based on the surgical techniques used for DALK. For this review, we will consider a microperforation as a small perforation that, in general, did not lead to consistent anterior chamber collapse as compared to a macroperforation, in which a sizeable tear or gap in DM (usually 0.5 mm or more in length) resulted in persistent, complete collapse of the anterior chamber despite the use of air or balanced salt solution to reform the chamber [4, 5].

### 2.2. Outcomes

#### 2.2.1. Primary Outcome: Best-Corrected Distance Visual Acuity (BCVA)

LogMAR BCVA was obtained from studies and converted to LogMAR if not presented in this format. If data were presented as a median with a range, the mean was estimated according to the formula by Hozo et al. [9].

#### 2.2.2. Secondary Outcomes

1. Endothelial cell density (ECD)
2. Graft rejection
3. Graft survival
4. Other complications include a double anterior chamber requiring rebubbling and conversion to PK

As studies were expected to have varying follow-ups, data were presented for the latest follow-up visit.

### 2.3. Search Methods for Identifying Studies

A PubMed and Google Scholar search for all relevant articles using the keywords deep anterior lamellar keratoplasty (DALK), DALK + perforation/DALK + rupture, published up to 9 April, 2025. Microsoft Excel (Microsoft 365, USA) spreadsheet was used to outline complications and outcomes. Titles and abstracts resulting from the search were assessed by two reviewers (SM and RM), and a full-text review of the selected abstracts was carried out. Data were presented as weighted mean values and pooled standard deviation or as a range, as applicable. The data were collated from all included studies and compared between eyes with DALK and DM perforation versus DALK without DM perforation using an unpaired *t*-test. A *p*-value of < 0.05 was considered statistically significant.

## 3. Results

**Figure 1:**
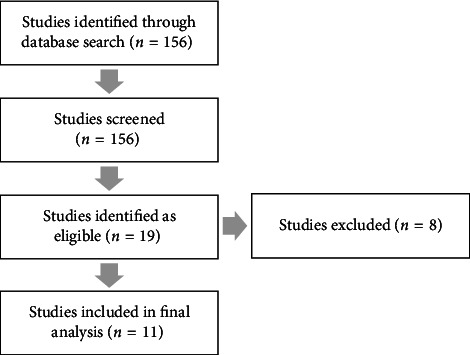
Flowchart of recruitment of studies.

One hundred and fifty-six studies were identified as part of the search criteria, which led to 19 studies that evaluated outcomes of DALK, including those with DM perforation, as identified within their abstracts. Eleven studies [3–5, 8, 11–16] were included in the analysis (Figure 1 and Table 1), while eight studies [7, 10, 17–22] were excluded due to a lack of subanalysis of DM perforations amongst their DALK cohort, no postoperative visual acuity data or conversion to PK in all cases of DM perforation [7, 10, 17–22]. All studies were retrospective case series. Most studies had a fixed follow-up of 12 months with shorter intervals prior to this (*n* = 5); others reported a range of follow-up (*n* = 2; range: 6–54 months). The mean age was 36.34 ± 11.97 years, with a total of 357 eyes across all eligible studies. Two hundred and thirty-six DALK surgeries resulted in microperforations, 106 in macroperforations and 8 in undefined perforations. Common indications for surgery included keratoconus (*n* = 135), scars from infectious keratitis or hydrops episodes (*n* = 45), dystrophy (*n* = 9) and other ectasias, such as pellucid marginal degeneration (*n* = 4).

Cases of perforations were typically classified into macroperforations or microperforations. The microperforations were identified as a small perforation that caused an aqueous leak but did not lead to complete loss of the anterior chamber, as compared to a macroperforation, in which a large tear in the DM (usually 0.5 mm or more in length) resulted in the collapse of the anterior chamber despite the use of air or balanced salt solution to reform the anterior chamber [4, 5]. DM perforation most commonly occurred during the lamellar stromal dissection step (65.2%), followed by big bubble formation (13.3%) and suturing (7.6%).

### 3.1. Primary Outcome (BCVA)

The mean preoperative BCVA was 1.11 ± 0.36 logMAR (*n* = 334) and 1.13 ± 0.52 logMAR (*n* = 598) in perforation and nonperforation groups, respectively (*p*=0.53). Three of the studies included in the review did not provide preoperative BCVA data [8, 11, 15]. The mean postoperative BCVA at the 12–24 months follow-up period was 0.35 ± 0.37 logMAR (*n* = 357) and 0.39 ± 0.07 logMAR (*n* = 455) in those with and without DM perforation, respectively (*p*=0.02). Postoperative BCVA at the 3-month interval demonstrated worse acuity in eyes with DM perforations compared to those without (0.5 ± 0.8 logMAR vs. 0.3 ± 0.2 logMAR, *p*=0.02). However, at the 12-month follow-up period in the same study [12], the BCVA became similar amongst the groups and was not statistically significantly different. This was the case in all studies that compared outcomes to eyes without DM perforations in their cohorts [4, 5, 14]. Of the studies with DM perforations, only one study [5] compared postoperative BCVA between microperforations and macroperforations at 12 months. However, there was no statistically significant difference (0.39 ± 0.23 vs. 0.45 +±0.39, respectively, *p*=0.60).

### 3.2. Secondary Outcomes

#### 3.2.1. Graft Rejection and Survival

Of the 11 studies included in the analysis, nine [3–5, 8, 11, 13, 15, 16, 23] commented on graft rejection rate. The reported rejection rate across these was 1.25% versus 1.6% in DALK with and without perforation (*p*=0.66). Primary graft failure was 0% in the majority (*n* = 9), and two studies [4, 12] reported a rate of 6.93% and 8%. The overall primary graft failure rate for DM perforations was 2.5% across 357 eyes, compared to 3.74% across 562 eyes without DM perforations (*p*=0.31).

The average preoperative ECD was 2404.24 ± 382.18 cells/mm^2^. ECD was measured postoperatively in eight eligible studies [8, 11–14, 16], all with a follow-up of at least 6 months after surgery. The average postoperative ECD was 1662.41 ± 319.16 cells/mm^2^ in patients with DM perforations and 2274.21 ± 506.02 cells/mm^2^ in the nonperforated group (*p* < 0.01). Two studies demonstrated that ECD was significantly lower in the DM perforation groups compared to those without DM perforations [12, 14]. One study [5] showed a significantly lower postoperative ECD at more than 12 months in cases with macroperforation compared to those in microperforation (1377.4 ± 366.5 vs. 1756.4 ± 232.5, *p* < 0.05). However, cases that were converted to PK demonstrated significantly lower ECD when compared to cases with microperforation (1756.4 ± 232.5 vs. 1222.6 ± 193.9, *p* < 0.01), although not when compared to cases with macroperforation (1377.4 ± 366.5, *p*=0.15) [5].

Only two studies evaluated survival rates [4, 12]. In the study by Huang et al. [4], no significant difference was found between graft survival rates in eyes with and without DM perforations. This also extended to rates of graft failure and graft rejection. However, an older study by Den et al. [12] found that DM perforation significantly affected graft survival via endothelial decompensation, which occurred in three eyes. Of note, two of the three eyes were macroperforations, and all cases of macroperforations were complicated by pseudochamber formation.

### 3.3. Complications and Conversion to PK

Across all studies, DM detachment and double anterior chamber/pseudochamber formation were a very common postoperative complication encountered after DALK with DM perforation. Such cases are usually managed with rebubbling in the postoperative period. All studies reported the rebubbling rates, except for one study [8], where there were no cases that required rebubbling (*n* = 8). The rates ranged from 0% to 100%, with a weighted mean of 22.8%.

Studies by Goweida et al. [11], Den et al. [12] and Hashish et al. [14] excluded patients requiring conversion to PK. For the remainder of the studies [3–5, 8, 13, 15, 16, 23], the mean rate of conversion to PK was 4.23%. Other common documented complications included pupillary block (*n* = 8), elevated IOP (*n* = 8), Urretz Zavalia syndrome (*n* = 6) and interface haze (*n* = 3).

Notably, in the study by Seifelnasr et al. [5], one case amongst the PK conversion group developed graft failure, compared to none amongst all DALK groups, both with and without DM perforation. However, they did not find any statistically significant differences in the number of eyes with postoperative graft failure between the groups (*p*=0.33). Seifelnasr et al. [5] found that three PK-converted cases developed at least one episode of graft rejection (all were endothelial), compared to none in the other three DALK groups.

## 4. Discussion

DM perforation remains one of the most common complications of DALK surgery. It is generally accepted that earlier in the surgeon's learning curve, there will be a higher rate of DM perforation and conversion to PK. However, there is now increasing evidence that DALK can be completed and managed even in the context of DM perforation. This helps to facilitate the benefits that come with retaining the host endothelium, such as reduced rates of rejection and improved graft survival. It is also well known that postoperative high IOP and cataracts are more common in PK compared to those in DALK [1]. This provides the rationale for DALK over PK, especially in younger keratoconic patients [1]. Our narrative review aims to report on the outcomes of DALK with DM perforations in the current literature and compare them to outcomes in uncomplicated DALK cases.

The primary outcome of our review was BCVA. Comparing all the studies using weighted means, we noted a small, statistically significant difference in the BCVA between the two groups, with better BCVA in the perforation group. However, this is likely a result of the total weighted mean and standard deviation in the analysis being heavily influenced by one study [23] due to its reported good BCVA outcomes and large patient numbers; therefore, these results should be interpreted with caution. Only one study [5] directly compared postoperative BCVA at 12 months between microperforations and macroperforations. However, it found no statistically significant difference, again suggesting that any greater interface haze induced by a macroperforation does not become clinically significant at the 12-month point. However, this was not the case after longer periods of follow-up, particularly at the 12-month time point [5]. This suggests that comparable visual outcomes can be achieved, even in the context of DM perforation. The same study also found no statistically significant difference in postoperative BCVA between DALK with DM perforations (both micro and macro) and those who underwent DALK, which converted to PK, at 12 months [5]. Of the four studies comparing DALK with and without perforation, there was no statistically significant difference in postoperative BCVA between eyes with and without DM perforations at the 1-year follow-up period [4, 5, 12, 14]. In multiple studies, there was often worse BCVA in DALK eyes with DM perforation in the more immediate postoperative period (range: 1 week to 3 months). This may be attributed to DM wrinkles or increased interface haze, including that from a double anterior chamber [24, 25]. Previous studies have shown that the long-term postoperative visual outcomes of DALK and PK are similar [17, 20]. A prior meta-analysis comparing DALK and PK cases found no significant difference in the BCVA of eyes undergoing DALK versus PK at 6- and 12-month post-surgeries [26].

Graft rejection, failure and endothelial cell count were the secondary outcomes in our review. Although not statistically significant, we observed fewer rejections and failures in the perforation group compared to those in the nonperforation group. Of the 11 studies included in our review, 9 [3–5, 8, 11, 13, 15, 16, 23] commented on the graft rejection rate. The reported rejection rate across these was 1.25% in DALK with perforation. It is worth noting that all cases of rejection amongst DM perforations occurred in one study, whereby subepithelial rejection was detected in four cases between the third and fourth years postoperatively, all at least 6 months after topical steroid cessation [23]. Of note, in one study [5], they did not demonstrate any episodes of rejection in either case of micro- or macroperforation. However, cases that were converted to PK had a 14.3% incidence of rejection. This suggests that the reduced rejection risk is maintained, even when a DM perforation occurs intraoperatively. As suggested by one case report [3], this may be explained by the host migration of endothelial cells across sites of DM rupture, thereby providing immunological security. In a meta-analysis of outcomes of DALK vs. PK in keratoconus [26], the graft rejection rate was significantly higher in the PK group than in the DALK group (OR = 0.28; 95% CI 0.15 to 0.50; *p* < 0.001); however, with regard to graft failure, they did not find any significant difference (OR = 1.05; 95% CI 0.81 to 1.36; *p*=0.73). Primary graft failure was reported in all 11 studies, with a total rate of 2.5% across 357 eyes with perforation. It is noted that graft failure was only observed in 2 of the 11 studies. One of these studies was considerably larger than most other studies included in the analysis [4], while the other [12] had a significantly higher incidence of pseudochamber formation in the postoperative period compared to the other studies in the analysis. In one of the largest studies of 101 eyes with DM perforation [4], no significant difference in graft failure, graft rejection or graft survival was noted compared to cases without DM perforation. Interestingly, they also saw no difference between macro- and microperforations with regard to graft survival. Only one other study in the literature has examined graft survival following intraoperative DM perforations in DALK surgery [12]. In this study by Den et al. [12], the rate of endothelial decompensation was significantly higher in those with DM perforations compared to those without at 6 months. This difference may be attributed to their series having an older cohort of patients and a higher incidence of pseudochamber/double anterior chamber in the postoperative period (60%) compared to Huang et al. [4], which reported 38.6%. A subanalysis of their data revealed that endothelial decompensation occurred more frequently in cases with macroperforations than in those with microperforations [12]. This is similarly reflected in another study, which demonstrated a significantly lower post-op ECD at > 12 months in cases with macroperforation compared to those in microperforation (1377.4 ± 366.5 vs. 1756.4 ± 232.5, *p* < 0.05) [5].

In our review, the weighted mean postoperative ECD in the perforation group was significantly lower. Interestingly, in one study, DALK cases that were converted to PK demonstrated significantly lower ECD when compared to cases with microperforation, though not when compared to cases with macroperforation. Notably, a study by Den et al. [12] reported lower ECD compared to all other studies. However, they had higher age in this study group with a large standard deviation. This low cell density in their study [12] could be attributed to the increased incidence of postoperative pseudochamber in 60% of cases with DM perforation, and not all was managed with rebubbling, with three cases undergoing endothelial decompensation despite air/gas tamponade. It is well known that after PK, endothelial cell loss occurs at a significantly higher rate compared to the healthy, naïve eye. In contrast, another major advantage of DALK is the low rate of endothelial cell loss; therefore, this further justifies reducing conversion to PK during DALK where possible. In a randomised multicentre trial, the mean ECD in patients undergoing DALK and PK at 12 months was 1936 ± 643 cells/mm^2^ and 1966 ± 321 cells/mm^2^, respectively [20]. However, endothelial cell loss was not significantly different between DALK and PK when cases with DM perforation were included in their analysis. The clinical significance of the reduced ECD in DM rupture cases is less certain, as studies to date have not shown inferior outcomes with regard to BCVA and overall graft survival, although long-term follow-up data are lacking. In addition, while the mean ECD in PK groups was more reduced than in DALK groups, a meta-analysis demonstrated no statistically significant difference between the central corneal thickness (CCT) of the two groups [26]. Similar results were reported by Kettesy et al. [27], who found no correlation between ECD and CCT.

Across all studies, DM detachment and double anterior chamber/pseudochamber formation were common postoperative complications encountered after DALK with DM perforation. Such cases are usually managed with rebubbling in the postoperative period. Our review findings confirm this, with a total of 22.4% of patients undergoing rebubbling in the postoperative period across all studies in our review. In three studies [5, 12, 14], DM detachment and rebubbling rates were compared between micro- and macroperforations. Seifelnasr et al. found that almost half of the DALK cases with DM macroperforation developed DM detachment (eight cases, 53.3%), while six cases (27.3%) of the microperforation group developed DM detachment [5]. Single rebubbling was needed in 13 cases, and one case required rebubbling three times. In the study by Den et al., all six cases with macroperforation developed DM detachment with pseudochamber formation, compared to 9 out of 19 (47.4%) cases with microperforation [12]. Again, in the study by Hashish et al., six cases (60%) with microperforation developed DM detachment with pseudochamber formation required rebubbling, compared to three (100%) of cases with macroperforation [14].

We found DM perforation commonly occurred during the lamellar stromal dissection step (65.19%) followed by big bubble formation (13.3%). In one of the largest studies by Huang et al., 53.5% of DM perforations occurred when the big bubble technique was used and 43.5% when manual dissection was performed. They also found no significant difference in graft survival between perforated cases performed via the big bubble technique and those done using the manual technique.

Our review is limited by the relatively small number of studies eligible, as few studies specifically focused on patients with DM perforations or separated them from uncomplicated DALKs during analysis of their results. Another limitation is that all studies in our review were retrospective case series. However, DALK is performed infrequently across the globe, as the major indication of DALK is keratoconus, and its progression is very well arrested by crosslinking. Also, given that the cohort of patients who underwent DALK with perforation is likely to remain small conducting prospective studies with a sufficiently large sample size would always be challenging. Only one study [5] compared microperforation with macroperforation and presented the subanalysis of primary and secondary outcomes. Moreover, there is no consensus on the definition of macro and microperforations, with different surgeons and studies having differing thresholds of conversion to PK. Furthermore, our review had to include studies from different centres and surgeons with varying surgical skills and experience, which may introduce some biases. In addition, as our review consisted of case reports and series, it was not possible to plot forest plots. Finally, it is difficult to predict the risk of perforation in DALK-eligible cases, as it appears to be highly dependent on the surgeon, technique and pathology. It was not possible to establish a relationship between the risk of DALK perforation and aetiological indication for DALK.

The question of whether surgeons should convert to PK when encountering DM perforation during DALK was controversial, but this review highlights that, wherever possible, continuing with DALK after perforation provides good outcomes. While further refinement of surgical technique and procedure is needed to reduce this common complication, the findings of this narrative review suggest that micro- and even macroperforations of DM can be safely managed without conversion to PK, with comparable outcomes to those of uncomplicated DALK. Further research exploring the size and location of DM perforation on outcomes is warranted to enable clearer criteria to help surgeons make better decisions about converting to PK when faced with this complication.

## Figures and Tables

**Table 1:** Included studies.

Study	Year	Number of eyes	Big bubble/manual dissection	Perforation	Mean age	Preop BCVA (logMAR)	Postop BCVA at 12 months (logMAR)	Primary failure (%)	Rejection (%)	Rebubbling/anterior chamber formation (%)	Endothelial cell density (cells/mm^2^)
Khattak et al. [10]	2023	119	100% BB	93 micro, 26 macro	35.6 ± 0	0.89	0.18 (24 months)	0	3	16	1748 ± 161
Huang et al. [4]	2019	101	53.5% BB, 43.5% manual	79 micro, 15 macro	38.3 ± 19.2	1.2 ± 0.6	0.55 ± 0.6	6.93	0	23.7	N/A
Seifelnasr et al. [5]	2023	58	56.9% BB, 43.1% manual	22 micro, 36 macro	33.21 ± 11.61	1.36 ± 0.34 (micro), 1.25 ± 0.29 (macro)	0.39 ± 0.23 (micro), 0.45 +±0.39 (macro)	0	0	22.4	1756.4 ± 232.5 (micro), 1377.4 ± 366.5 (macro)
Leccisotti [8]	2021	12	100% BB	All macro	26.8 ± 11.4	N/A	0.32 ± 0.09	0	0	75	1830 ± 299.7
Jhanji et al. [7]	2007	25	100% manual	19 micro, 6 macro	52.2 ± 18.3	1.2 ± 0.9	0.3 ± 0.9	8	N/A	12	924 ± 422
Hozo et al. [9]	2023	13	100% BB	10 micro, 3 macro	28.73 ± 9.79	1.50 ± 0.0	0.30 ± 0.12	0	N/A	75	1732.62 ± 539.52
Goweida [11]	2007	8	100% BB	Undefined	37.3 ± 11.4	N/A	0.1 ± 0.8	0	0	0	2179
Den et al. [12]	2018	16	87.5% BB, 12.5% manual	4 macro, 12 micro	29.6 ± 6.4	1.07 ± 0.3	0.28 ± 0.09	0	0	12.5	2267 ± 160
Kodavoor et al. [13]	2012	1	100% BB	1 micro	23 ± 0	1.8	0.3	0	0	0	2005
Hashish et al. [14]	2019	3	66.66% BB, 33.33% manual	3 macro	31 ± 0	N/A	0.17 ± 0.29	0	0	33.3	N/A
Ashena and Nanavaty [3]	2020	1	100% manual	1 macro	64 ± 0	0.3	0.17	0	0	100	N/A

*Note:*BCVA = best-corrected distance visual acuity, Micro = microperforation, and Macro = macroperforation.

Abbreviation: BB = big bubble.

## Data Availability

The data that support the findings of this study are available from the corresponding author upon reasonable request.
